# Dysfunction of Salivary Glands, Disturbances in Salivary Antioxidants and Increased Oxidative Damage in Saliva of Overweight and Obese Adolescents

**DOI:** 10.3390/jcm9020548

**Published:** 2020-02-17

**Authors:** Anna Zalewska, Agnieszka Kossakowska, Katarzyna Taranta-Janusz, Sara Zięba, Katarzyna Fejfer, Małgorzata Salamonowicz, Paula Kostecka-Sochoń, Anna Wasilewska, Mateusz Maciejczyk

**Affiliations:** 1 Experimental Dentistry Laboratory, Medical University of Bialystok, 15-222 Bialystok, Poland; a_kossak@op.pl; 2 Department of Pediatrics and Nephrology, Medical University of Bialystok, 15-222 Bialystok, Poland; katarzyna.taranta@wp.pl (K.T.-J.); annwasil@interia.pl (A.W.); 3 Students Scientific Club “Biochemistry of Civilization Diseases” at the Department of Hygiene, Epidemiology and Ergonomics, Medical University of Bialystok, 15-222 Bialystok, Poland; sarap.zieba@gmail.com; 4 Conservative Dentistry Department, Medical University of Bialystok, 15-222 Bialystok, Poland; katarzyna.fejfer@gmail.com (K.F.); wrobmalg@gmail.com (M.S.); p.kosta@wp.pl (P.K.-S.); 5 Department of Hygiene, Epidemiology and Ergonomics, Medical University of Bialystok, 15-222 Bialystok, Poland

**Keywords:** oxidative stress, antioxidants, saliva, salivary biomarkers, obesity

## Abstract

Obesity is inseparably connected with oxidative stress. This process may disturb the functioning of the oral cavity, although the effect of oxidative stress on salivary gland function and changes in the qualitative composition of saliva are still unknown. Our study is the first to evaluate salivary redox homeostasis in 40 overweight and obese adolescents and in the age- and gender-matched control group. We demonstrated strengthening of the antioxidant barrier (↑superoxide dismutase, ↑catalase, ↑peroxidase, ↑uric acid, ↑total antioxidant capacity (TAC)) with a simultaneous decrease in reduced glutathione concentration in saliva (non-stimulated/stimulated) in overweight and obese teenagers compared to the controls. The concentration of the products of oxidative damage to proteins (advanced glycation end products), lipids (malondialdehyde, 4-hydroxynonenal) and DNA (8-hydroxydeoxyguanosine) as well as total oxidative status were significantly higher in both non-stimulated and stimulated saliva as well as plasma of overweight and obese adolescents. Importantly, we observed more severe salivary and plasma redox alterations in obese adolescents compared to overweight individuals. In the study group, we also noted a drop in stimulated salivary secretion and a decrease in total protein content. Interestingly, dysfunction of parotid glands in overweight and obese teenagers intensified with the increase of BMI. We also showed that the measurement of salivary catalase and TAC could be used to assess the central antioxidant status of overweight and obese adolescents.

## 1. Introduction

Overweight/obesity is a social problem worldwide, characterized by an increase in body weight that results in excessive accumulation of fat [1]. In recent years, we have observed a steady growth in the frequency of overweight and obesity, observed not only among adults, but also children and adolescents [2]. This results from various genetic, environmental and economic (easy access to cheap and highly calorific food) factors as well as evolutionary conditioning (sedentary lifestyle, low physical activity, low energy expenditure) [1,3]. According to the latest WHO report, nearly 41 million children under 5 years of age are overweight or obese [4]. Interestingly, studies have shown that about 40% of overweight children will continue to gain weight during the puberty period, and about 80% of these obese teenagers will remain obese as adults [5]. Although obesity rates are higher in developed countries, more overweight or obese children live in developing countries, and this trend also applies to European countries.

It has been shown that obesity is associated with an increase in oxidative stress (OS). OS is a condition characterized by disturbed balance between the amount of reactive oxygen species (ROS) produced by the body and activity/concentration of antioxidants responsible for neutralizing ROS [6]. ROS are chemically reactive molecules which, when unbalanced, lead to oxidative modifications of proteins, lipids, carbohydrates and nucleic acids, resulting in obesity-related complications.

It should be emphasized that obesity has been recognized as a major underlying factor in the pathogenesis of serious OS-related health problems, such as hyperlipidemia [7], insulin resistance [8], hypertension [9], type 2 diabetes [10], cardiovascular diseases [7] and certain types of cancer [11]. Overweight/obesity has been shown to adversely affect the condition of the oral cavity, including salivary gland function [12,13,14]. The pathogenesis of salivary gland lesions in the course of obesity is not fully understood, although the influence of OS is emphasized. It has been demonstrated that adult morbid obesity is associated with disorders of antioxidant systems [15,16] and oxidative damage to salivary proteins, lipids, and DNA [17], while bariatric treatment generally lowers the levels of salivary oxidative damage. However, it does not rescue antioxidant capacity of non-stimulated and stimulated saliva [16,17].

Recently, more and more attention has been paid to the use of saliva in laboratory medical diagnostics, particularly in connection with pediatric diseases, as the collection of saliva samples is non-invasive and thus acceptable to children. It has been shown that proteins and other substances are transported to saliva from blood via the passive process of diffusion, ultrafiltration and active transport. The concentrations of numerous substances in saliva can be correlated with their concentrations in blood plasma, allowing for the use of saliva as an alternative diagnostic material. Moreover, the use of oxidative stress biomarkers is proposed in diagnosing patients with obesity due to the observed changes in enzymatic and non-enzymatic antioxidant levels as well as accumulation of protein and lipid oxidation products in plasma and saliva of obese patients [16,17].

There have been numerous studies on OS in saliva and blood of overweight and obese adults [15,16,17,18], whereas no research has been conducted to evaluate salivary redox markers and their usefulness in diagnosing adolescents with excessive body weight. Therefore, the aim of our work is to evaluate antioxidant systems as well as oxidative modifications of proteins and lipids in non-stimulated and stimulated saliva of overweight and obese adolescents.

## 2. Materials and Methods

### 2.1. Patients

The study was approved by the Ethics Committee at the Medical University of Bialystok, Poland (permission number R-I-002/43/2018). After explaining the purpose and methodology of the study to patients and their parents, written informed content was obtained from each parent/legal guardian.

Our study included adolescents aged 11–18: overweight with BMI z-score ≤ + 1 + 2 < SD (*n* = 20, 10 teenagers) and obese with BMI z-score ≥ + 2 SD (*n* = 20, 10 teenagers).

The control group consisted of adolescents (*n* = 40, 20 teenagers) with normal body weight (BMI z-score < –1 + 1 <, matched by age and gender to the study group.

The adolescents included in the study group were recruited in the Department of Pediatrics and Nephrology of the Medical University of Bialystok, during routine follow-up visits, after performing a dental and biochemical blood test and meeting the conditions for inclusion/exclusion to the study.

The adolescents included in the control group were recruited during dental follow-up visits in the Children’s Outpatient Dentistry Clinic (Specialist Dental Centre of the Medical University of Bialystok), initially based on BMI index and a health survey. Then, after obtaining the written consent of participants and their legal guardians, biochemical blood tests were performed. If the participants met the conditions for inclusion/exclusion in the study, they were finally included in the control group.

Patients from the study and control group were qualified by the same experienced pediatrician (K.T.J.) as well as a pediatric dentist (A.Z.). The control group consisted of patients of the Children’s Outpatient Dentistry Clinic because, in the Pediatrics and Nephrology Clinic, there were only a few healthy controls that met the inclusion criteria for the study.

Clinical data of the subjects are presented in Table 1.

Body weight, height and head and chest circumferences were measured by standard methods. BMI was calculated as weight (kg) divided by the square of height (m^2^). BMI z-scores that reflect the standard deviation score (SD) for age- and gender-appropriate BMI distribution, were calculated according to the LMS method [19], using reference values from the WHO study [20]. Based on the international norms from the World Health Organization for age- (with an accuracy of 1 month) and gender-specific BMI, BMI cut-offs for children over 5 years of age were the following: overweight–BMI z-score ≤ + 1 + 2 < SD; obesity–BMI z-score ≥ + 2 SD [21].

The inclusion criteria were: adolescents of both sexes (in the case of girls, those who had had menarche) with full permanent dentition. On the day of material collection, the teenage girls were in the first phase of their menstrual cycle.

Patients with deciduous and mixed dentition, with gingivitis (gingival index, GI > 0.5) and pathological changes in the oral cavity mucosa were excluded from the study. Negative general medical history was a necessary factor to qualify for the experiment. The questionnaire completed by the patients included: infectious, autoimmune and metabolic diseases (type 2 diabetes) as well as hypertension, insulin resistance and diseases of respiratory, cardiovascular, digestive, genitourinary and coagulation systems. The exclusion criteria also covered inappropriate behavior and/or refusal to cooperate with the examiner. At least 3 months before the study, patients and the healthy controls had not taken any oral non-steroidal anti-inflammatory drugs, glucocorticosteroids, vitamins, other supplements, or antibiotics. The participants were non-smokers and did not drink alcohol more frequently than once a month.

### 2.2. Blood Collection

Blood was collected on an empty stomach, during routine examinations in the case of adolescents from the control group, and for the study groups: during admission to the Pediatric and Nephrology Clinic of the Medical University of Bialystok. Blood was collected in the amount of 10 mL using an S-Monovette^®^ EDTA K3 tube (Sarstedt, Nümbrecht, Germany). After collection, the blood was centrifuged (3000 g, 10 min, 4 °C). No hemolysis was observed in any of the obtained plasma samples. Blood cell mass was rinsed 3 times with 0.9% NaCl, and then underwent osmotic lysis using 50 mM cold phosphate buffer (pH 7.4) 1:9 (v/v) [17]. To prevent sample oxidation, 0.5 M BHT (Sigma-Aldrich, Saint Louis, MO, USA; 10 µl/ml blood) was added to the plasma and red blood cell lysate [17]. Plasma and blood lysate were frozen (–82 °C). The samples were stored deep-frozen for no longer than 6 months.

### 2.3. Saliva Collection

Non-stimulated and stimulated saliva was collected by the spitting method between 7 and 8 a.m., one day after admission to the Pediatrics and Nephrology Clinic of the UMB or during a routine dental check-up. The time since the last meal, tooth-brushing and taking medications was at least 10 h. Samples were collected in a separate room to ensure comfort for the subjects. After rinsing the mouth with water, participants spat out non-stimulated saliva accumulated at the bottom of the oral cavity for 15 min. Stimulated saliva was collected after a 5-min break. Stimulation was performed by dropping 20 µl of 2% citric acid on the tongue every 20 s for 5 min. Both types of saliva were collected in test tubes placed on ice. To prevent sample oxidation, 0.5 M BHT (Sigma-Aldrich, Saint Louis, MO, USA; 10 µl/ml blood) was added to the saliva. After collecting the samples, the volume of saliva was measured in a calibrated pipette with accuracy of 100 µL and saliva flow rate was estimated. Saliva samples were centrifuged (3000 g, 20 min, 4 °C, MPW 351, MPW Med. Instruments, Warsaw, Poland) and then frozen (–82 °C). Frozen samples were stored for no longer than 6 months.

In each of the obtained saliva samples, the concentration of transferrin was determined by the ELISA test to identify samples contaminated with blood. Transferrin was not detected in any of the saliva samples (data not shown).

### 2.4. Dental Examination

Immediately after saliva collection, dental examination was performed by the selected dentists (K. F., M. S., P. K.-S.) in artificial light, using a mirror, an explorer and a periodontal probe (WHO, 621) in accordance with the WHO criteria. The dental examination included the evaluation of DMFT (decayed, missing, filled teeth) and GI (gingival index). The interrater reliability for DMFT was *r* = 0.92, and for GI was *r* = 0.94.

### 2.5. Biochemical Determination

The performed assays included: antioxidant enzymes (salivary peroxidase (Px), EC 1.11.1.7, catalase (CAT), EC 1.11.1.6 and superoxide dismutase (SOD), EC 1.15.1.1), non-enzymatic antioxidants (reduced glutathione (GSH), uric acid (UA) and albumin), redox status (total oxidant status (TOS), total antioxidant capacity (TAC) and oxidative stress index (OSI)), advanced glycation end products (AGE), malondialdehyde (MDA), 4-NHE-protein adduct (4-HNE) and 8-hydroxy-D-guanosine (8-OHdG). All results were standardized to mg of total protein. The total protein content was determined using the bicinchoninic acid (BCA) method and bovine serum albumin (BSA) as a standard (Thermo Scientific PIERCE BCA Protein Assay (Rockford, IL, USA).

In the saliva samples, we analyzed all redox biomarkers. In erythrocytes, antioxidant enzymes were assayed, while in the blood plasma we evaluated non-enzymatic antioxidants, redox status and oxidative damage products as well as interleukin-6 (IL-6) concentration. All assays were performed in duplicate samples (TOS in triplicate samples), and the absorbance/fluorescence of the samples was measured with an Infinite M200 PRO Multimode Microplate Reader (Tecan).

### 2.6. Antioxidant Enzymes

The activity of salivary peroxidase (Px, E.C. 1.11.1.7) was determined colorimetrically according to Mansson-Rahemtulla et al. [22] based on the reduction of 5,5’-dithiobis-(2-nitrobenzoic acid) (DTNB) to thionitrobenzoic acid, which then reacted with thiocyanate anions (SCN^-^) formed as a result of potassium thiocyanate (KSCN) oxidation by Px. The absorbance was measured at a 412 nm wavelength. The activity of catalase (CAT, E.C. 1.11.1.6) was determined by the colorimetric method described by Aebi [23], based on the measurement of the hydrogen peroxide (H_2_O_2_) decomposition rate in phosphate buffer at pH 7.0. The absorbance was measured at 240 nm wavelength. One unit of CAT activity was defined as the amount of the enzyme that decomposes 1 mM H_2_O_2_ for 1 min. The activity of superoxide dismutase-1 (SOD, E.C. 1.15.1.1) was determined colorimetrically according to Misra and Fridovich [24] based on the measurement of cytoplasmic activity of the SOD subunit in the reaction of inhibiting the oxidation of epinephrine to adrenochrome at a 320 nm wavelength. It was assumed that one unit of SOD activity inhibits epinephrine oxidation in 50%.

### 2.7. Non-Enzymatic Antioxidants

The concentration of reduced glutathione (GSH) was determined colorimetrically based on DTNB reduction to 2-nitro-5-mercaptobenzoic acid under the influence of GSH contained in the assayed samples [25]. Absorbance changes were measured at 412 nm wavelength. Uric acid (UA) concentration was determined by the colorimetric method using a set of ready-made reagents (QuantiChrom TM Uric Acid Assay Kit DIUA-250, BioAssay System Harward, CA, USA). The method is based on the reaction of 2,4,6-tripyridyl-s-triazine with iron (3+) ions in the presence of UA contained in the samples. Changes in the absorbance of the resulting complex were measured at a 590 nm wavelength.

### 2.8. Redox Status

Total antioxidant capacity (TAC) was determined colorimetrically as described by Erel [26], based on the measurement of the ability to neutralize the radical cation ABTS ^∙+^ [2,2-azino-bis-(3-ethylbenzothiazoline-6-sulfonate)] under the influence of antioxidants contained in the tested samples. Changes in the absorbance of ABTS ^∙+^ solution were measured at a 660 nm wavelength. Total oxidant status (TOS) was determined using the colorimetric method described by Erel [27], based on the oxidation of iron (2+) ions to iron (3+) ions in the presence of oxidants contained in the sample, followed by the detection of Fe^3+^ ions by xylene orange. TOS concentration was calculated from the standard curve for hydrogen peroxide and presented in nM H_2_O_2_ equivalent/mg total protein. TOS determination was performed in triplicate samples. The oxidative stress index (OSI) was presented as the quotient of TOS to TAC and expressed in % [28,29].

### 2.9. Products of Oxidative Damage to Proteins and Lipids

The content of protein advanced glycation end products (AGE) was determined fluorimetrically by the method described by Kalousová et al. [30] based on the measurement of fluorescence of furyl-furanyl-imidazole (FFI), carboxymethyl lysine (CML), pyraline and pentosidine at the excitation wavelength 350 nm and emission wavelength 440 nm. To determine the AGE content, the samples were diluted in PBS buffer (0.02 M, pH 7.0) in a volume ratio of 1:5, and mixed thoroughly [31]. AGE content was determined in duplicate samples and expressed in fluorescence arbitrary units AFU/mg total protein. The concentration of malondialdehyde (MDA) was determined colorimetrically using thiobarbituric acid (TBA) [32]. The MDA reaction with TBA produces a colored adduct with the maximum absorption at 535 nm wavelength. The concentrations of 4-HNE and 8-OHdG were assessed by ELISA with commercial sets (Cell Biolabs, Inc. San Diego, CA, USA; USCN Life Science, Wuhan, China, respectively), following the manufacturer’s instructions included in the package.

### 2.10. Statistical Analysis

Statistical analysis of the results was performed using GraphPad Prism 8 and Microsoft Excel 16.16.12 for MacOS. The D’Agostino–Pearson test and Shapiro–Wilk test were used to assess the distribution of the results. Individual groups were compared using the analysis of variance (ANOVA) followed by Tukey’s honest significant difference test (Tukey’s HSD test). The multiplicity adjusted p value was also calculated. Correlations between redox biomarkers were assessed based on the Pearson correlation coefficient. The results were presented as mean and standard deviation (SD) using tables or graphs. Diagnostic usefulness of the redox biomarkers was evaluated by means of receiver operating characteristic (ROC) analysis. Statistical significance was assumed at *p* ≤ 0.05.

The number of subjects was determined based on our previous experiment, assuming that the power of the test would equal 0.9.

## 3. Results

### 3.1. General Characteristics

Stimulated secretion was significantly lower in the group of overweight and obese adolescents compared to the controls (↓40%, *p* < 0.0001; ↓51%, *p* < 0.0001, respectively). Teenagers with obesity secreted considerably less saliva after stimulation than their overweight peers (18%, *p* = 0.004). The secretion of non-stimulated saliva did not differ significantly between the study groups as well as in comparison with the control group (Table 2).

Total protein concentration in stimulated saliva of overweight and obese adolescents was significantly lower than in the control group (↓33%, *p* = 0.0001; 40%, *p* < 0.0001, respectively). Protein concentration in non-stimulated saliva did not differ significantly between the study groups and compared to the control group (Table 2).

**Figure 1:**
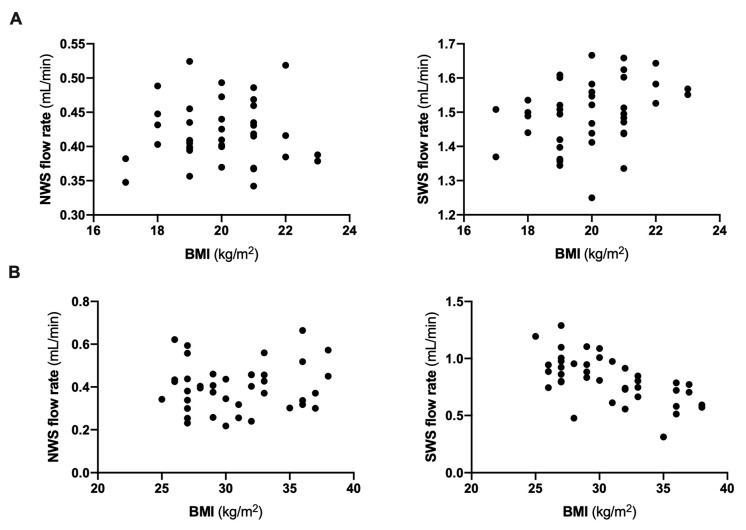
Scatter plots for BMI and non-stimulated and stimulated salivary flow rate in healthy children (**A**) as well as overweight and obese adolescents (**B**). BMI- body mass index; NWS- non-stimulated whole saliva; SWS- stimulated whole saliva.

Scatter plots for BMI and NWS/SWS flow rate are presented in Figure 1.

There were no significant differences in the dental indexes DMFT and GI between the controls and the groups of overweight and obese adolescents (Table 2).

### 3.2. Enzymatic Antioxidants

The activity of SOD in non-stimulated and stimulated saliva of overweight adolescents was significantly higher than in the control group of adolescents with normal weight (↑60%, *p* < 0.001; ↑48%, *p* = 0.002, respectively). Similar significant differences were observed in the group of obese adolescents, in whom SOD activity in non-stimulated and stimulated saliva was significantly higher than in the control group (↑125%, *p* < 0.001; ↑78%, *p* < 0.001, respectively). There were no differences in salivary SOD activity between overweight and obese subjects. SOD activity in erythrocytes of both overweight (↓59%, *p* < 0.001) and obese (↓58%, *p* < 0.001) adolescents was considerably lower than in erythrocytes of teenagers with normal body weight, and did not differ between the study groups.

The activity of CAT in non-stimulated saliva of obese adolescents was significantly higher compared to the controls (↑75%, *p* < 0.001) and overweight adolescents (↑75%, *p* < 0.001).

The activity of CAT in stimulated saliva of overweight (↑62%, *p* < 0.001) and obese (↑90%, *p* < 0.001) adolescents was significantly higher than in the control group. CAT activity in erythrocytes of obese teenagers was considerably lower than in the control group (↓49%, *p* < 0.001) and in overweight adolescents (↓38%, *p* = 0.02).

**Figure 2:**
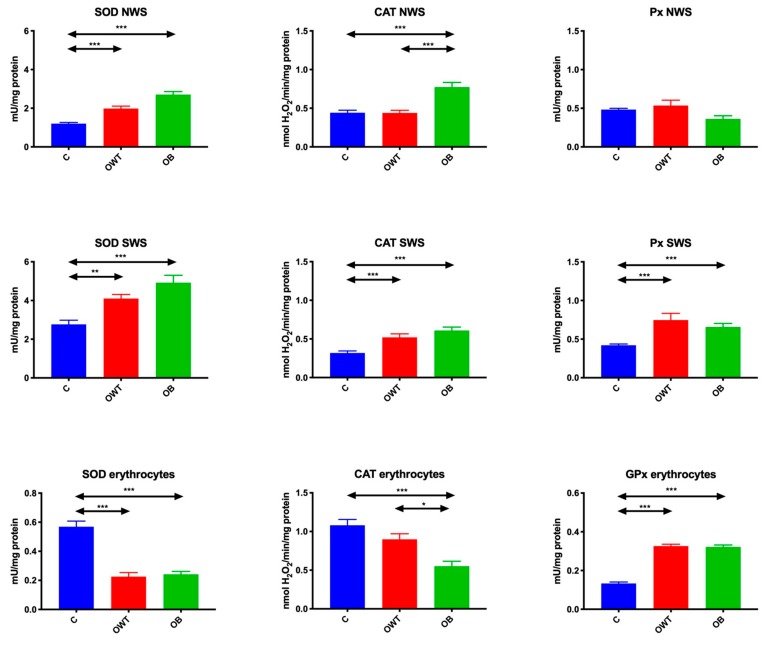
Enzymatic antioxidants in overweight and obese adolescents as well as healthy controls. C- control, CAT- catalase, NWS- non-stimulated whole saliva, OB- obese, OWT- overweight, Px- salivary peroxidase, SOD- superoxide dismutase, SWS- stimulated whole saliva. Differences statistically significant at: * *p* < 0.05, ** *p* < 0.005, *** *p* < 0.0005.

The activity of Px in non-stimulated saliva did not differ between the study groups and the controls. Px activity in stimulated saliva and erythrocytes of overweight (↑78%, *p* < 0.001; ↑153%, *p* < 0.001, respectively) and obese (↑57%, *p* < 0.001; ↑153%, *p* < 0.001, respectively) adolescents was significantly higher than in saliva and erythrocytes of the control group (Figure 2).

### 3.3. Non-Enzymatic Antioxidants

The concentration of GSH in non-stimulated and stimulated saliva of overweight (↓47%, *p* < 0.001; ↓26%, *p* = 0.005, respectively) and obese (↓65%, *p* < 0.001; ↓54%, *p* < 0.001, respectively) adolescents was significantly lower than in the control group. However, the GSH concentration in overweight adolescents was significantly higher only in stimulated saliva compared to obese subjects (↑38%, *p* < 0.001). Plasma GSH concentration in obese adolescents was significantly lower than in the control group (↓29%, *p* < 0.006).

**Figure 3:**
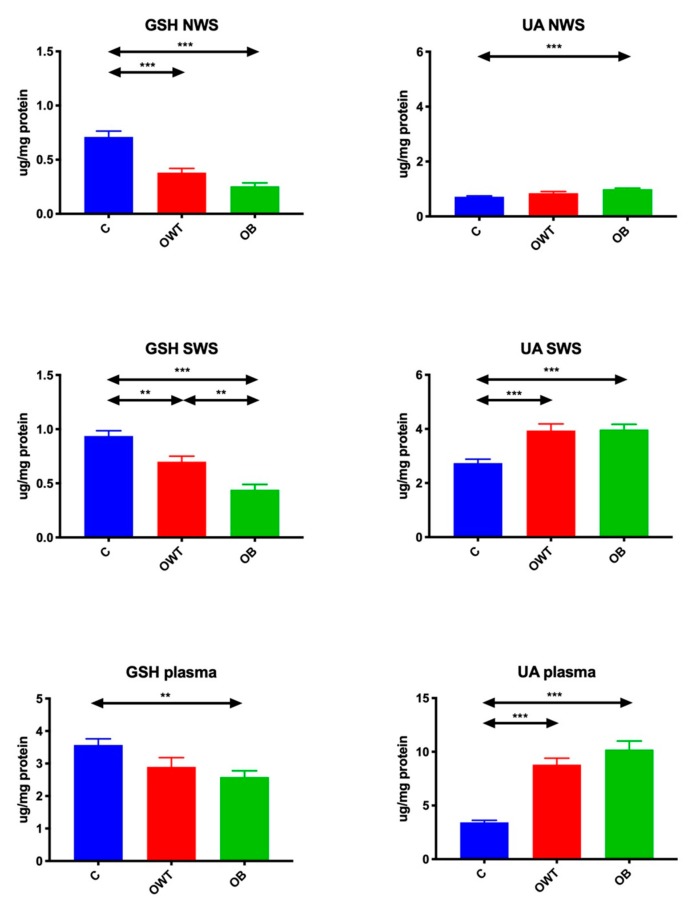
Non-enzymatic antioxidants in overweight and obese adolescents as well as healthy controls. C- control, GSH- reduced glutathione, NWS- non-stimulated whole saliva, OB- obese, OWT- overweight, SWS- stimulated whole saliva, UA- uric acid. Differences statistically significant at: ** *p* < 0.005, *** *p* < 0.0005.

The concentration of UA in non-stimulated saliva of obese adolescents was significantly higher than in the controls (↑37%, *p* < 0.001). UA concentration in stimulated saliva of overweight and obese adolescents was considerably higher than in the control group (↑157%, *p* < 0.001; ↑198%, *p* < 0.001, respectively). Plasma UA concentration in overweight and obese adolescents was significantly elevated compared to their peers with normal body weight (↑43%, *p* < 0.001; ↑45%, *p* < 0.001, respectively) (Figure 3).

### 3.4. Redox Status

In overweight and obese adolescents, TAC in non-stimulated (↑110%, *p* < 0.001; ↑122%, *p* < 0.001, respectively) and stimulated (↑62%, *p* < 0.001; ↑56%, *p* < 0.001, respectively) saliva as well as plasma (↑61%, *p* < 0.001; ↑75%, *p* < 0.001, respectively) was considerably higher than in the control group.

TOS in non-stimulated (↑113%, *p* < 0.001; ↑288%, *p* < 0.001, respectively) and stimulated (↑115%, *p* < 0.001; ↑170%, *p* < 0.001, respectively) saliva as well as plasma (↑103%, *p* < 0.001; ↑97%, *p* ≤ 0.001, respectively) of overweight and obese adolescents was significantly raised compared to the control group. TOS in non-stimulated (↑129%, *p* < 0.001) and stimulated (↑25%, *p* = 0.001) saliva of obese teenagers was considerably higher than in their overweight peers.

**Figure 4:**
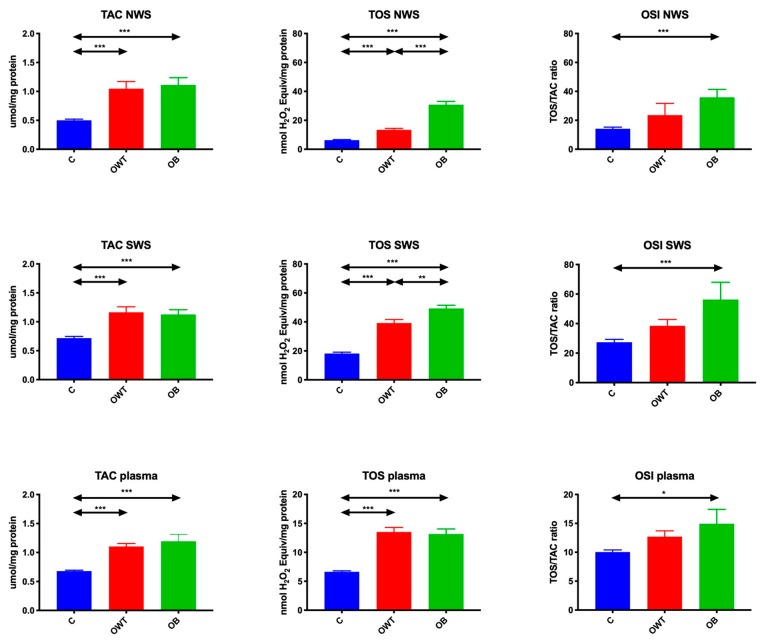
Redox status in overweight and obese adolescents as well as healthy controls. C- control, NWS- non-stimulated whole saliva, OB- obese, OSI- oxidative stress index, OWT- overweight, SWS- stimulated whole saliva, TAC- total antioxidant capacity, TOS- total oxidative status. Differences statistically significant at: * *p* < 0.05, ** *p* < 0.005, *** *p* < 0.0005.

OSI in non-stimulated and stimulated saliva as well as plasma in obese adolescents was significantly higher than in the controls (↑153%, *p* < 0.001; ↑105%, *p* = 0.001; ↑48%, *p* = 0.01, respectively) (Figure 4).

### 3.5. Oxidation Products

AGEs in non-stimulated and stimulated saliva as well as plasma of both overweight (↑281%, *p* < 0.001; ↑209%, *p* < 0.001; ↑203%, *p* < 0.001, respectively) and obese (↑347%, *p* < 0.001; ↑423%, *p* < 0.001; ↑244%, *p* < 0.001, respectively) adolescents were significantly higher compared to the saliva of the control group. Only AGEs in stimulated saliva of obese adolescents were considerably higher than in overweight adolescents (↑69%, *p* < 0.001).

MDA in non-stimulated and stimulated saliva as well as plasma in both overweight (↑43%, *p* < 0.001; ↑63%, *p* = 0.001; ↑41%, *p* < 0.001, respectively) and obese (↑43%, *p* < 0.001; ↑79%, *p* < 0.001; ↑55%, *p* < 0.001, respectively) teenagers were significantly higher than in saliva of control group adolescents.

**Figure 5:**
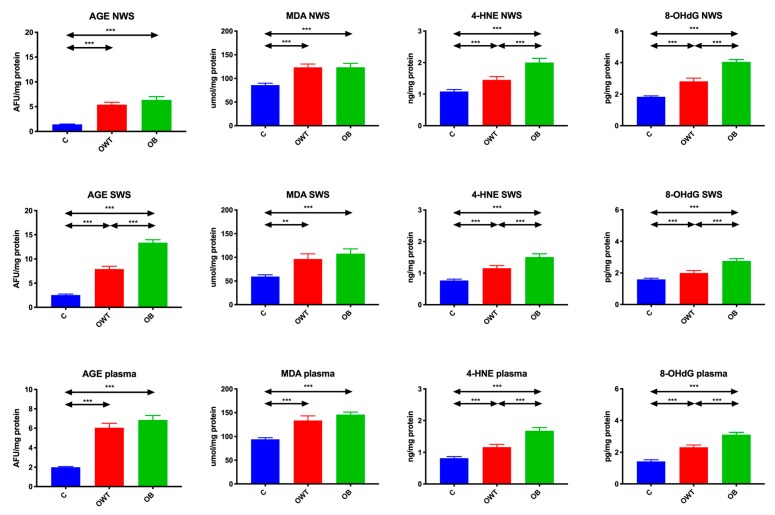
Protein, lipid, and DNA oxidation products in overweight and obese adolescents as well as healthy controls. AGE- advanced glycation end products, C- control, MDA- malondialdehyde, NWS- non-stimulated whole saliva, OB- obese, OWT- overweight, SWS- stimulated whole saliva, 4-HNE- 4-hydroxynoneal protein adduct, 8-OHdG- 8-hydroxy-D-guanosine. Differences statistically significant at: ** *p* < 0.005, *** *p* < 0.0005.

The concentration of 4-HNE was notably higher in non-simulated and stimulated saliva as well as plasma of overweight (33% *p* = 0.01; 50% *p* < 0.001; 41% *p* = 0.003) and obese adolescents (84% *p* < 0.001; 95% *p* < 0.001; 104% *p* < 0.001) compared to the control group. The concentration of 4-HNE in non-simulated and stimulated saliva as well as plasma of obese adolescents was considerably higher than in overweight adolescents (37% *p* < 0.001; 50% *p* < 0.001; 43% *p* < 0.001). Similarly, the concentration of 8-OHdG in non-stimulated and stimulated saliva as well as plasma of both overweight (53% *p* < 0.001; 25% *p* = 0.04; 62% *p* < 0.001) and obese adolescents (121% *p* < 0.001; 73% *p* < 0.001; 118% *p* < 0.001) was significantly higher than in saliva and plasma of the controls. The 8-OHdG concentration in non-stimulated and stimulated saliva as well as plasma of obese teenagers was considerably higher than in their overweight peers (43% *p* = 0.001, 38% *p* = 0.007; 34% *p* < 0.001) (Figure 5).

### 3.6. ROC Analysis

The diagnostic utility of salivary redox parameters to differentiate children who are overweight from those who are obese is presented in Table 3. For this purpose, ROC curves were generated, and then the area under the curve (AUC) was calculated. Optimal cut-off values were determined for each parameter that ensured high sensitivity with high specificity. The maximum AUC value, from 0 to 1, is a parameter that determines the discriminatory power of the test.

**Figure 6:**
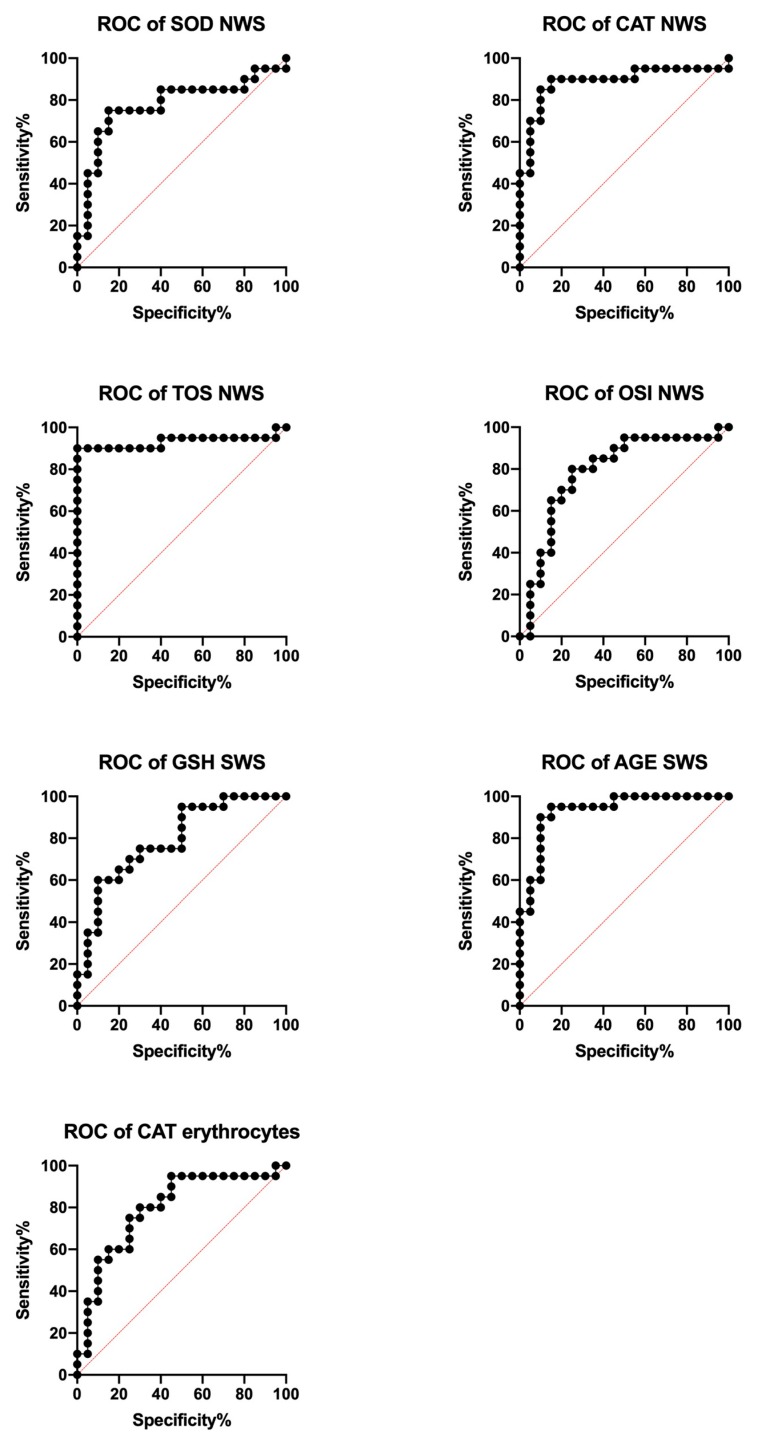
Area under the curve (AUC) of selected redox biomarkers in overweight and obese children. AGE- advanced glycation end products, CAT- catalase, GSH- reduced glutathione, NWS- non-stimulated whole saliva, OSI- oxidative stress index, SOD- superoxide dismutase, SWS- stimulated whole saliva, TOS- total oxidative status, UA- uric acid, 4-HNE- 4-hydroxynoneal protein adduct,.

Particular attention should be paid to SOD, CAT, TOS and OSI in NWS, GSH and AGE in SWS, and CAT in erythrocytes—the AUC of which is close to 1.0, which differentiates overweight adolescents from obese ones (Figure 6).

### 3.7. Correlations

**Figure 7:**
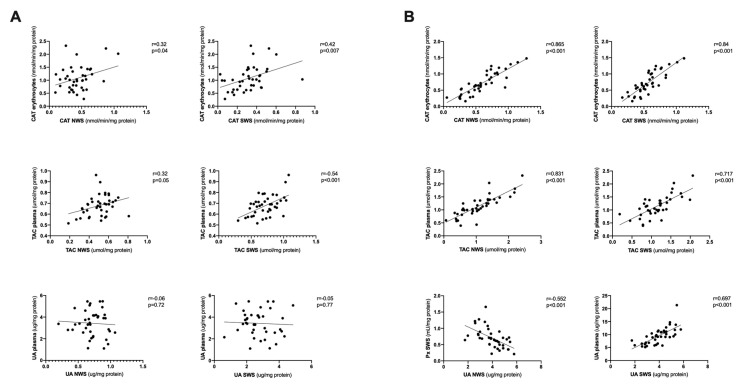
Saliva–blood correlations of the analyzed redox biomarkers in healthy controls (**A**) as well as overweight and obese adolescents (**B**). CAT- catalase, NWS- non-stimulated whole saliva, SWS- stimulated whole saliva, TAC- total antioxidant capacity, UA- uric acid.

We showed a positive correlation between erythrocyte and salivary CAT and TAC in overweight and obese adolescents. We also demonstrated a positive correlation between UA content in plasma and non-stimulated/stimulated saliva of the study group patients (Figure 7B). However, we did not observe a saliva–blood correlation of UA in healthy children and adolescents (Figure 7A).

**Figure 8:**
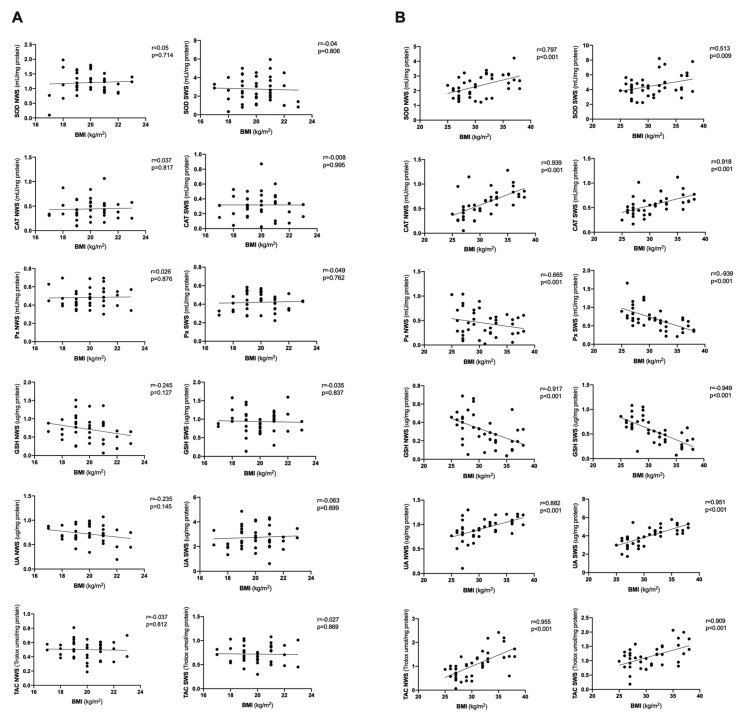
Correlations between BMI and salivary antioxidants in healthy children (**A**) as well as overweight and obese adolescents (**B**). BMI- body mass index, CAT- catalase, GSH- reduced glutathione, NWS- non-stimulated whole saliva, Px- salivary peroxidase, SOD- superoxide dismutase, SWS- stimulated whole saliva, TAC- total antioxidant capacity, UA- uric acid.

**Figure 9:**
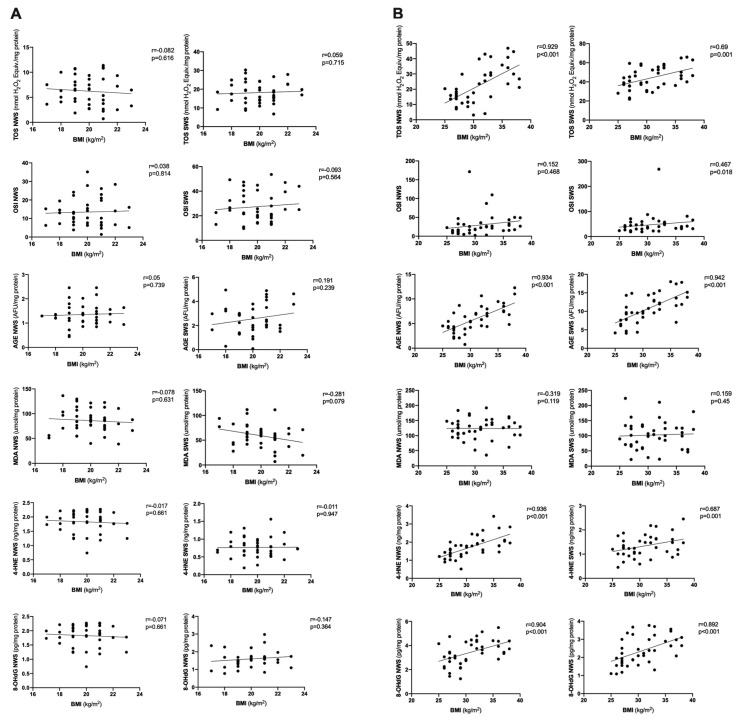
Correlations between BMI and salivary oxidative damage in healthy children (**A**) as well as overweight and obese adolescents (**B**). BMI- body mass index, AGE- advanced glycation end products, MDA- malondialdehyde, NWS- non-stimulated whole saliva, OSI- oxidative stress index, SWS- stimulated whole saliva, TOS- total oxidative status, 4-HNE- 4-hydroxynoneal protein adduct, 8-OHdG- 8-hydroxy-D-guanosine.

Correlations between BMI and salivary redox biomarkers and presented in Figure 8 and Figure 9. Interestingly, BMI correlates with most salivary antioxidants and oxidative damage products but only in the study group.

## 4. Discussion

This publication is the first to analyze redox balance in the saliva of overweight and obese adolescents. Generally, we demonstrated disturbances in the activity/concentration of antioxidants as well as oxidative stress in non-stimulated and stimulated saliva of both examined groups compared to their peers with normal body weight, with a higher intensity of oxidative modifications in the saliva of obese adolescents.

Excessive body weight is characterized by chronic (low-grade) inflammation with permanently elevated OS. It has been demonstrated that adipose tissue induces the synthesis of proinflammatory cytokines, such as TNFα, IL-1 and IL-6, which increase the generation of ROS and nitrogen radicals by macrophages and monocytes. ROS production promotes inflammation and expression of molecules as well as growth factors by redox-sensitive transcription factors, mainly NF-κB and the NADPH oxidase pathway [33,34]. The inefficiency of antioxidant systems, observed in the plasma of obese individuals [16], entails oxidative damage to cellular components and development of obesity-related complications.

It has been shown that obesity results in the dysfunction of salivary glands as well as changes in salivary flow and composition [15,16,17,35]. As saliva is essential for maintaining appropriate functions of the body, such as swallowing, chewing, carbohydrate digestion, healing of the oral mucosa and tooth enamel remineralization, it is not surprising that excessive body weight increases the risk of gingivitis [36], periodontitis [37], caries [38,39] and inflammatory changes in the oral cavity mucosa [40]. Recently, a significant influence of OS has been increasingly emphasized in explaining the pathogenesis of salivary gland lesions in the course of overweight/obesity in adults [16,17,41]. To the best of our knowledge, there have been no studies evaluating redox balance in the oral cavity of overweight and obese adolescents.

The study by Brown et al. [42] demonstrated that failure of antioxidant systems is related to the duration of obesity. Considering that our study covered adolescents aged 11 to 18 with relatively short overweight/obesity history (4/4.3 years, respectively, data not shown), it is not surprising that salivary and plasma TAC (the sum of both enzymatic and non-enzymatic antioxidants) as well as the content of enzymatic antioxidants were elevated in both non-stimulated and stimulated saliva of overweight and obese adolescents. Therefore, the higher activity of enzymatic antioxidants may be an expression of a highly effective antioxidant barrier that has not been exhausted in oxidative stress conditions. On the other hand, a significant increase in salivary TAC and enzymatic antioxidants as well as, generally, plasma biomarkers (despite the decline in SOD activity, which we explain further) can be considered as a positive adaptative response to the increased ROS generation (↑TOS in plasma as well as non-stimulated and stimulated saliva of both study groups). It was demonstrated that decreased activity of antioxidant enzymes in erythrocytes, accompanying increased plasma TAC, is likely to result from cell damage due to an inflammatory process and leakage of enzymes into the extracellular space [43]. On the other hand, it may result from the use of enzymes in the process of ROS control, or from inactivation of enzymes by free radicals [44].

Interestingly, we found a significant positive correlation between erythrocyte and salivary CAT and TAC in overweight and obese adolescents, which suggests that these salivary parameters could assess the general antioxidant status of overweight and obese adolescents.

Uric acid constitutes 40% of the antioxidant potential of saliva [45]; however, it has been found that at high concentrations it can induce and intensify oxidative damage [46]. Obesity has been demonstrated to increase UA concentration by reducing its renal secretion and accumulating metabolites for UA production [47], which was confirmed by our study. We observed an increase in uric acid concentration both in plasma and stimulated saliva of overweight and obese adolescents as well as in non-stimulated saliva of the latter. The negative correlation between the concentrations of UA and protein in non-stimulated saliva of overweight and obese adolescents, as well as the negative correlation between UA content and Px activity in stimulated saliva, suggest that in both groups UA shifts salivary redox balance towards oxidation and does not oxidize hydroxyl or peroxyl radicals, preventing OS. Moreover, we demonstrated a positive correlation between UA levels in plasma and stimulated saliva of overweight and obese subjects. Considering that plasma UA is a strong predictor of future development of type 2 diabetes [48], measurements of salivary UA may be useful in assessing the risk of this disease.

Although changes in the activity/concentration of antioxidants or ROS concentration may suggest redox imbalance, they are not sufficient to determine the existence and extent of OS. The most reliable determinants of oxidative stress are increased concentrations of oxidative damage products [49,50]. There are numerous markers of oxidative damage to biomolecules; in our study, we assessed 4-HNE protein adducts, MDA, AGE and 8-OHdG. These are only few selected markers, which should be taken into account when interpreting the presented results. The use of other OS indicators could change our observations and conclusions.

Our research showed, however, that the overproduction of free radicals exceeds the capabilities of antioxidant systems of the examined adolescents at the central level as well as in both salivary glands, which was observed as increased concentration of oxidative modification products in plasma and non-stimulated as well as stimulated saliva. It should be noted that intensified oxidative modifications reveal a certain tendency (AGE and MDA in plasma and non-stimulated saliva; MDA in stimulated saliva) or are significantly higher (4-HNE, 8-OHdG in plasma and stimulated and non-stimulated saliva, and AGE in stimulated saliva) in obese adolescents compared to their overweight peers. The obtained results prove that oxidative damage occurred in the salivary glands of overweight adolescents, and was more severe in obese subjects. We noted a significant increase of TOS in non-stimulated and stimulated saliva in obese adolescents compared to the overweight group, but observed no such dependence for TAC and antioxidant enzymes (except CAT in non-stimulated saliva). In our opinion, this is a worrying phenomenon and may be evidence of the beginning of subclinical inefficiency of antioxidant mechanisms, leading to the boost of oxidative damage to the salivary glands of obese adolescents.

We demonstrated that in both non-stimulated and stimulated saliva of overweight and obese patients, GSH levels were significantly decreased and considerably lower in the stimulated saliva of patients with obesity compared to overweight ones. Our results suggest that, GSH is the first to be used up, and perhaps forms the first line of defense against free radicals, which can be easily explained. The main function of GSH is maintaining thiol groups of proteins in a reduced state, which is often necessary for preserving the functional activity of these proteins. It has been shown that the most probable primary object of ROS attack is proteins, and, according to this theory, fatty and nucleic acids are protected by proteins and therefore undergo oxidation at a later stage [51]. A mediator of biomolecule damage in cells is the hydroxyl radical (OH**^.^**) [52]. It was shown that the percentage share of primary substrates of the OH**^.^** reaction is 75% proteins, 21% lipids, and 4% DNA, which is related to the specificity of the mechanism of OH**^.^** radical production in the Fenton reaction [52].

It is worth mentioning that the stimulation of saliva secretion activates parotid glands, while at rest the main source of saliva is the submandibular glands. Therefore, it was assumed that any disturbances in secretion/composition of non-stimulated saliva reflects disturbances of the submandibular gland function, and disturbed secretion/composition of stimulated saliva indicates disturbed activity of parotid glands [53].

The ability of salivary glands of overweight and obese adolescents to secrete saliva at rest was similar to the results of their peers of normal weight. Protein secretion by submandibular glands of the examined adolescents also did not differ from the control group of normal weight. In relation to the stimulated salivary flow, overweight and obese subjects showed reduced salivary secretion compared to the control group, which is consistent with the results of Modéer et al. [38]. Moreover, the dysfunction of parotid glands intensified with the increase in BMI, as obese adolescents produce significantly less stimulated saliva than their overweight peers, and in 8 obese individuals we recorded the salivary flow value of 0.7 mL/min, which is classified as hyposalivation. Modéer et al. [38] claimed that BMI SDS may be a potential factor to exclude subjects with reduced flow of stimulated saliva. Protein concentration in SWS of overweight and obese adolescents was also reduced compared to the controls. The decrease in stimulated saliva secretion and protein concentration is most probably caused by steatosis of the parotid (but not submandibular) glands observed in obese patients, which decreases the number of secretory units (acini and ducts) [54,55]. It can also be assumed that obesity-related inflammatory milieu (up-regulation of proinflammatory cytokines, ROS), similarly to Sjögren’s syndrome [56], activates metalloproteinases, thus disrupting the stromal tissue. This phenomenon may disturb the neurotransmission between the residual neural network and residual secretory units, as well as inhibit the response of follicular cells [57,58].

Our study showed reduced salivary production and enhanced oxidative stress in overweight and obese teenagers. However, in obese children, alterations in salivary gland function are more severe than those who are overweight. This is also reflected in the redox status of saliva (greater disturbances in the antioxidant barrier) as well as higher severity of oxidative damage in children with obesity vs. overweight.

## 5. Conclusions

Obese and overweight adolescents present impaired systemic and salivary oxidative status in contrast to their normal weight peers.

Both parotid and submandibular salivary glands lose the ability to maintain redox balance at the level observed in the control group, which was shown by an increased level of oxidized biomolecules. However, redox equilibrium in our study was more disturbed in the saliva and plasma of obese adolescents compared to overweight subjects.

Excess of adipose tissue and deficiency of GSH are the main factors responsible for oxidative damage to the salivary glands.

Dysfunction of parotid glands in relation to salivary secretion deepens with the increase of BMI. Dysfunction of mechanisms responsible for protein synthesis/secretion observed at the overweight stage does not worsen with the increase of body weight in adolescents.

Determinations of salivary CAT and TAC could be used to assess the central antioxidant status of overweight and obese adolescents.

## Figures and Tables

**Table 1:** Clinical characteristics of patients and healthy controls.

	C *n* = 40	OWT *n* = 20	OB *n* = 20
Age (years)	16 ± 2.0	16 ± 1.9	15.8 ± 2.2
Sex (male/female) *n*	20/20	10/10	10/10
Weight (kg)	55 ± 3.1	65 ± 10 *	90 ± 21 *
Height (cm)	167 ± 4.5	163 ± 12	162 ± 14
BMI (kg/m^2^)	20 ± 1.5	28 ± 1.5 *	34 ± 2.7 *
cc BMI	50 ± 2.5	97 ± 1.2	99 ± 0.83
SDS BMI	0.5 ± 0.2	2.5 ± 0.28	3.9 ± 0.81
Systolic BP (mmHg)	109 ± 1.0	110 ± 1.2	111 ± 1.0
Diastolic BP (mmHg)	58 ± 4.2	73 ± 10	76 ± 9.8
WBC (thousand/μL)	5.8 ± 1.3	6.7 ± 2.2	6.7 ± 1.1
Hgb (g/dL)	13.9 ± 1.1	14 ± 1.2	14 ± 1.0
Hct (%)	42.0 ± 2.3	41 ± 3.3	42 ± 3.2
PLT (thousand/μL)	278 ± 46	294 ± 71	263 ± 48
sCre (mg/dL)	0.63 ± 0.1	0.64 ± 0.19	0.79 ± 0.56
Urea (mg/dL)	15.4 ± 3.6	24 ± 9.7 *	27 ± 13 *
HDL (mg/dL)	47 ± 2.2	52 ± 1.4	43 ± 8.1
LDL (mg/dL)	70 ± 5.2	87 ± 24	106 ± 21 *
Total cholesterol (mg/dL)	120 ± 9	132 ± 30	161 ± 39 *
TG (mg/dL)	68 ± 8	102 ± 75 *	139 ± 75 *
Glucose (mg/dL)	86 ± 5.3	86 ± 9.9	91 ± 5.4
eGFR (mL/min/1.73 m^2^)	132 ± 10	120 ± 42	122 ± 43
IL-6 (pg/mL)	1.7 ± 0.71	2.3 ± 0.74	3 ± 1.13

**Table 2:** Salivary flow rate, total protein and stomatological findings.

	C *n* = 40	O *n* = 20	OB *n* = 20
NWS (mL/min)	0.42 ± 0.05	0.39 ± 0.11	0.41 ± 0.11
SWS (mL/min)	1.5 ± 0.1	0.9 ± 0.2 *	0.74 ± 0.18 *^#^
TP NWS (μg/mL)	1291 ± 227	1167 ± 299	1139 ± 245
TP SWS (μg/mL)	986 ± 327	658 ± 208 *	598 ± 197 *
DMFT	6 ± 2	8 ± 3	8 ± 2
GI	0.1 ± 0.1	0.1 ± 0.15	0.1 ± 0.12

NWS- non-stimulated salivary flow rate, SWS- stimulated salivary flow rate, TP- total protein, C- control, OWT- overweight, OB- obese, DMFT= decay, missing, filling teeth, PBI- papilla bleeding index, GI- gingival index, * *p* < 0.05 vs. C; # *p* < 0.05 vs. OWT.

**Table 3:** Receiver operating characteristic (ROC) analysis to differentiate between overweight and obese children.

			NWS					SWS						Plasma/Erythrocytes				
Parameter	AUC	95% Confidence Interval	*p* Value	Cut-Off	Sensitivity (%)	Specificity (%)	AUC	95% Confidence Interval	*p* Value	Cut-Off	Sensitivity (%)	Specificity (%)	AUC	95% Confidence Interval	*p* Value	Cut-Off	Sensitivity (%)	Specificity (%)
*Antioxidants*																		
SOD (mU/mg protein)	0.7775	0.6222–0.9328	0.0027	>2.469	53.13	63.96	0.615	0.4360–0.7940	0.2134	>4.536	29.93	43.29	0.5325	0.3490–0.7160	0.7251	>0.2348	38.66	38.66
CAT (nmol H_2_O_2_/min/mg protein)	0.8875	0.7711–1.000	<0.0001	>0.5747	63.96	69.90	0.6675	0.4954–0.8396	0.0699	>0.5692	43.29	48.10	0.795	0.6515–0.9385	0.0014	<0.6559	53.13	53.13
Px/GPx (mU/mg protein)	0.6575	0.4818–0.8332	0.0884	<0.4494	43.29	38.66	0.5525	0.3675–0.7375	0.57	<0.6815	38.66	34.21	0.5	0.3167–0.6833	>0.9999	>0.3117	34.21	29.93
GSH (μg/mg protein)	0.7	0.5359–0.8641	0.0305	<0.3304	53.1	43.29	0.7925	0.6531–0.9319	0.0016	<0.5908	53.13	48.10	0.5875	0.4058–0.7692	0.3438	<2.823	43.29	34.21
UA (ng/mg protein)	0.67	0.5018–0.8382	0.0659	>0.8843	48.10	34.21	0.5075	0.3247–0.6903	0.9353	>3.912	29.93	29.93	0.6125	0.4345–0.7905	0.2235	>9.354	38.66	43.29
*Redox Status*																		
TAC (Trolox μmol/mg protein)	0.52	0.3370–0.7030	0.8287	>1.018	34.21	29.93	0.5175	0.3348–0.7002	0.8498	>1.202	29.93	38.66	0.5425	0.3511–0.7339	0.6456	>1.143	34.21	38.66
TOS (nmol H_2_O_2_ Equiv./mg protein)	0.9325	0.8344–1.000	<0.0001	>20.77	90	100	0.7425	0.5847–0.9003	0.0087	>45.71	70	80	0.55	0.3685–0.7315	0.5885	<14.48	34.21	29.93
OSI (TOS/TAC ratio)	0.7875	0.6377–0.9373	0.0019	>20.12	80	75	0.655	0.4836–0.8264	0.0935	>38.18	65	60	0.5525	0.3664–0.7386	0.57	<12.63	43.29	34.21
*Oxidative Damage*																		
AGE (AFU/mg protein)	0.5825	0.3937–0.7713	0.372	>6.116	38.66	48.10	0.9325	0.8547–1.000	<0.0001	>10.24	69.90	69.90	0.6175	0.4395–0.7955	0.2036	>6.360	38.66	38.66
MDA (μmol/mg protein)	0.5325	0.3480–0.7170	0.7251	>127.4	38.66	38.66	0.565	0.3828–0.7472	0.4819	>98.43	43.29	34.21	0.6625	0.4817–0.8433	0.0787	>143.0	53.13	48.10
4-HNE (ng/mg protein)	0.7625	0.6138–0.9112	0.0045	>1.787	65	70	0.705	0.5400–0.8700	0.0265	>1.268	70	70	0.81	0.6730–0.9470	0.0008	>1.386	75	80
8-OHdG (pg/mg protein)	0.8625	0.7452–0.9798	<0.0001	<3.440	80	80	0.795	0.6501–0.9399	0.0014	<2.226	75	80	0.8075	0.6761–0.9389	0.0009	>2.690	65	70

AGE- advanced glycation end products, CAT- catalase, GSH- reduced glutathione, MDA- malondialdehyde, NWS- non-stimulated whole saliva, OSI- oxidative stress index, Px- salivary peroxidase, SOD- superoxide dismutase, SWS- stimulated whole saliva, TAC- total antioxidant capacity, TOS- total oxidative status, UA- uric acid, 4-HNE- 4-hydroxynoneal protein adduct, 8-OHdG- 8-hydroxy-D-guanosine.
